# Supplementary material for: Trends in Urinary and Blood Cadmium Levels in U.S. Adults with or without Comorbidities, 1999–2018
Source: Nutrients. 2022 Feb 14;14(4):802. doi: 10.3390/nu14040802 (PMC8880632; doi:10.3390/nu14040802)
Supplement: Supplementary file 1 [file nutrients-14-00802-s001.zip › nutrients-1591935-supplementary.pdf]

Trends in Urinary and Blood Cadmium Levels in U.S. Adults with or without Comorbidities, 1999–2018

Figure S1. Flowchart of participant selection. 2

Figure S2. Trends in standardized geometric mean of blood cadmium levels by diabetes (A), coronary heart disease (B), stroke (C), and heart failure (D). 3

Figure S3. Trends in standardized geometric mean of urinary cadmium levels by diabetes (A), coronary heart disease (B), stroke (C), and heart failure (D). 4

Table S1. Standardized geometric mean of blood cadmium levels (in μg/L). 5

Table S2. Standardized geometric mean of urinary cadmium levels (in μg/g creatinine). 6

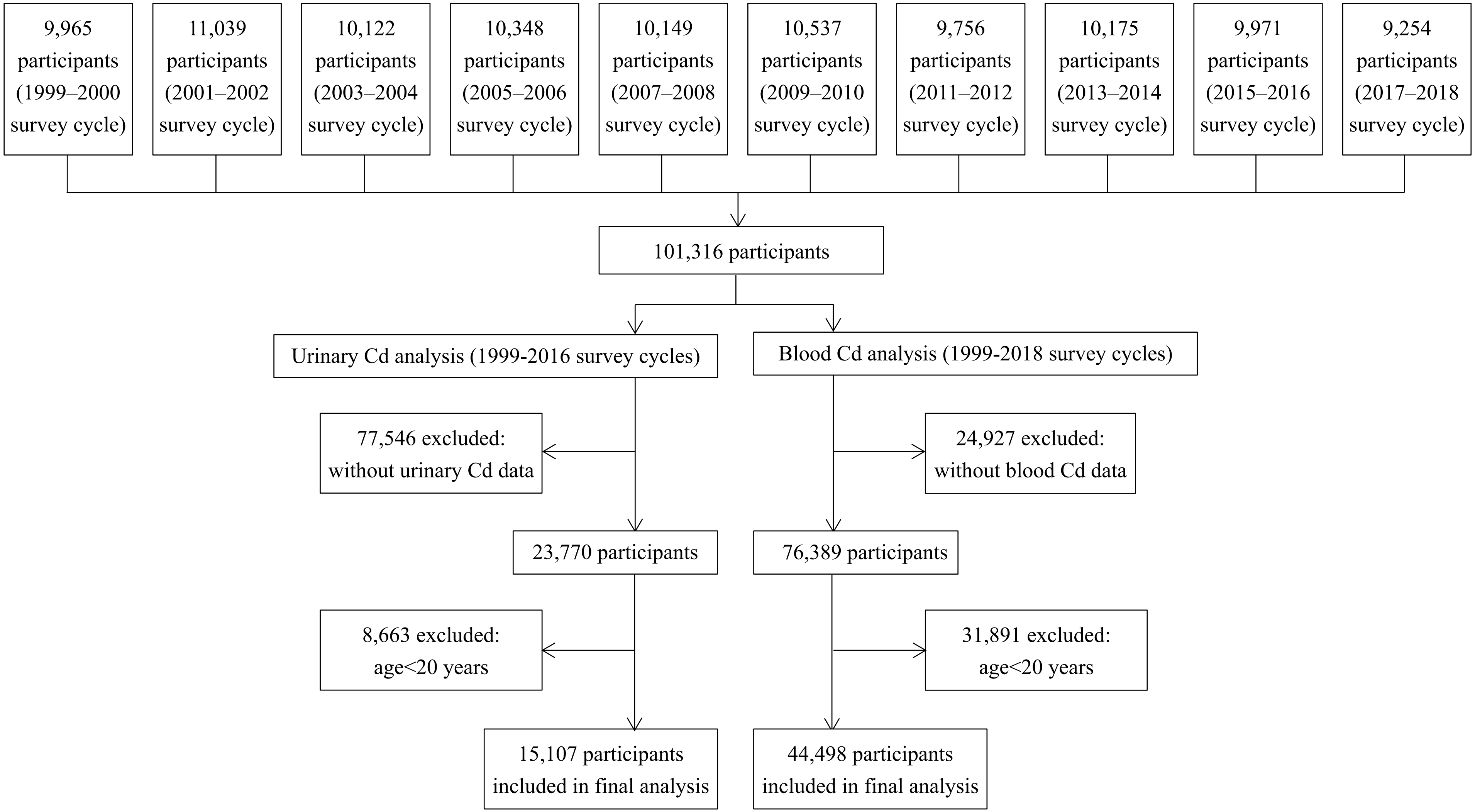

Figure S1. Flowchart of participant selection.

* Urinary cadmium data come from NHANES 1999-2016, and blood cadmium data come from NHANES 1999-2018.

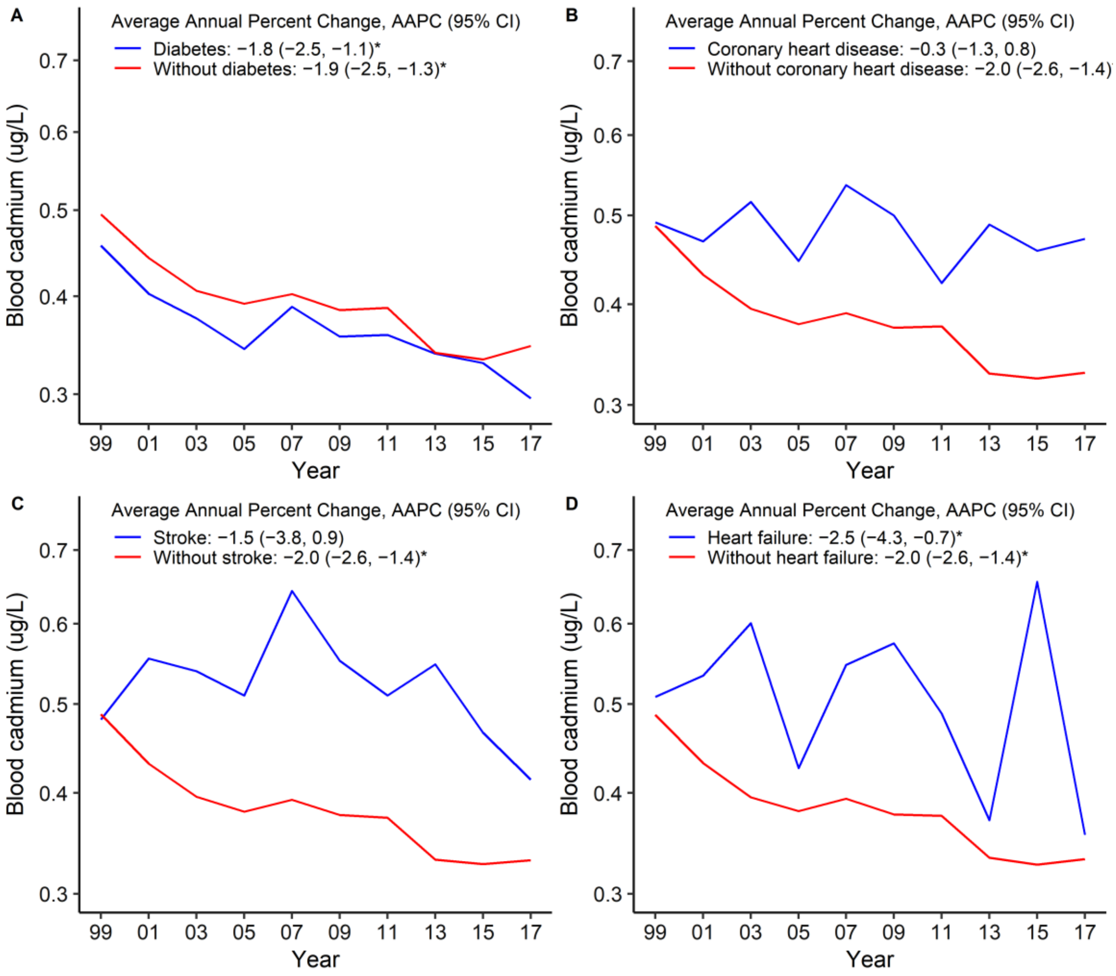

Figure S2. Trends in standardized geometric mean of blood cadmium levels by diabetes (A), coronary heart disease (B), stroke (C), and heart failure (D).

* The solid line indicated the age– and sex–standardized geometric mean of blood cadmium levels. Points indicate the change points (joinpoints) in trends detected by the Joinpoint regression model. The APPC is significantly different from zero at the α = 0.05 level. Data are from the U.S. National Health and Nutrition Examination Survey 1999-2018.

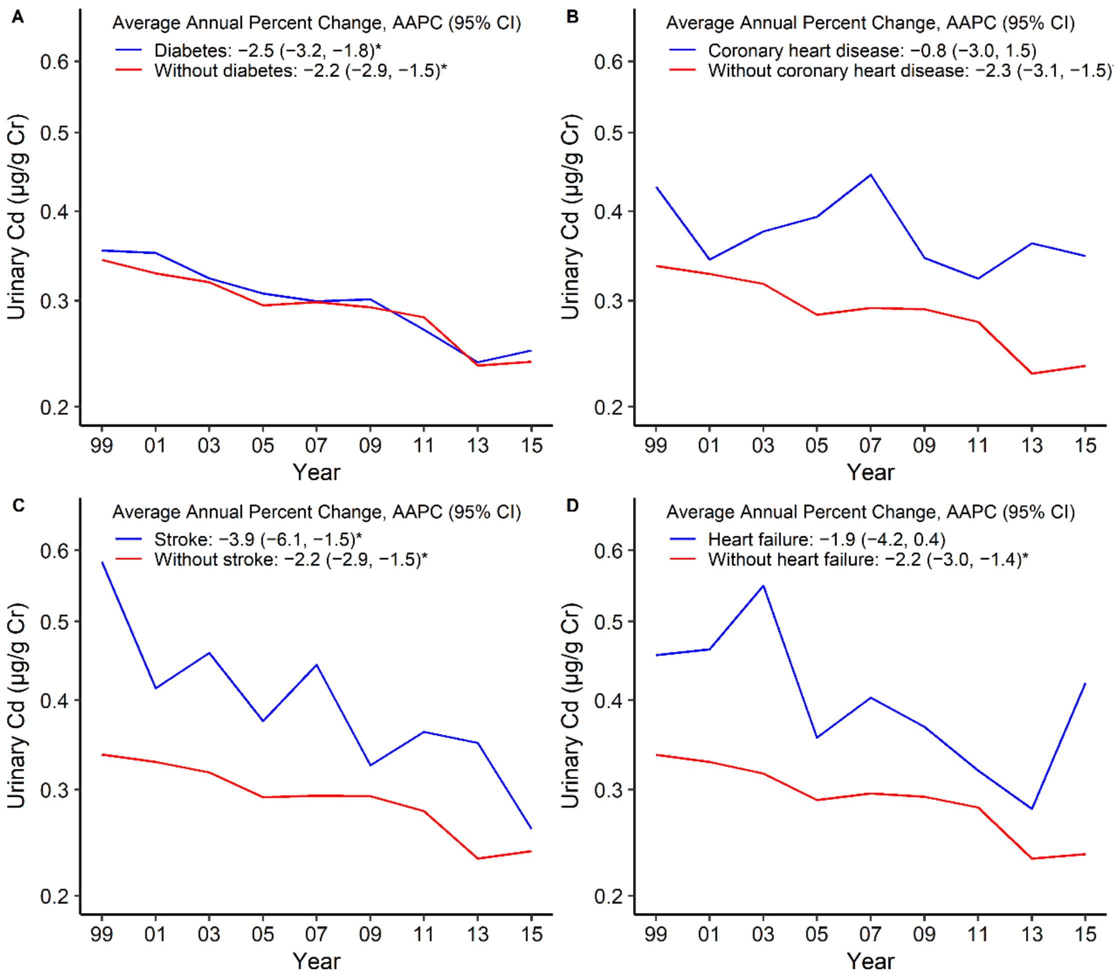

Figure S3. Trends in standardized geometric mean of urinary cadmium levels by diabetes (A), coronary heart disease (B), stroke (C), and heart failure (D).

* The solid line indicated the age– and sex–standardized geometric mean of urinary cadmium levels. Points indicate the change points (joinpoints) in trends detected by the Joinpoint regression model. The APPC is significantly different from zero at the $\alpha = 0.05$ level. Data are from the U.S. National Health and Nutrition Examination Survey 1999-2016.

Table S1. Standardized geometric mean of blood cadmium levels (in µg/L).

Group	1999–2000	2001–2002	2003–2004	2005–2006	2007–2008	2009–2010	2011–2012	2013–2014	2015–2016	2017–2018
Overall	0.49	0.43	0.40	0.38	0.40	0.38	0.38	0.34	0.33	0.33
Sex										
Men	0.49	0.42	0.39	0.37	0.38	0.36	0.35	0.30	0.31	0.30
Women	0.49	0.44	0.41	0.39	0.41	0.40	0.40	0.37	0.35	0.37
Smoking status										
Never smoker	0.38	0.32	0.28	0.27	0.28	0.27	0.28	0.24	0.25	0.25
Former smoker	0.46	0.40	0.36	0.34	0.35	0.35	0.34	0.33	0.32	0.30
Current smoker	0.97	0.95	1.02	0.98	1.03	0.94	1.02	0.91	0.88	0.95
Chronic kidney disease										
Yes	0.54	0.46	0.46	0.39	0.44	0.42	0.42	0.38	0.37	0.37
No	0.48	0.43	0.39	0.38	0.39	0.37	0.37	0.33	0.32	0.33
Hypertension										
Yes	0.49	0.44	0.41	0.40	0.41	0.40	0.39	0.36	0.36	0.35
No	0.49	0.44	0.40	0.38	0.40	0.37	0.38	0.33	0.32	0.34
Diabetes										
Yes	0.46	0.40	0.38	0.34	0.39	0.36	0.36	0.34	0.33	0.30
No	0.49	0.44	0.41	0.39	0.40	0.39	0.39	0.34	0.33	0.35
Cardiovascular disease										
Yes	0.50	0.50	0.54	0.43	0.51	0.52	0.44	0.46	0.47	0.42
No	0.49	0.43	0.39	0.38	0.39	0.37	0.37	0.33	0.32	0.33
Coronary heart disease										
Yes	0.49	0.47	0.52	0.45	0.54	0.50	0.42	0.49	0.46	0.47
No	0.49	0.43	0.40	0.38	0.39	0.38	0.38	0.33	0.32	0.33
Stroke										
Yes	0.48	0.56	0.54	0.51	0.64	0.55	0.51	0.55	0.47	0.41
No	0.49	0.43	0.40	0.38	0.39	0.38	0.37	0.33	0.33	0.33
Heart failure										
Yes	0.51	0.53	0.60	0.43	0.55	0.57	0.49	0.37	0.66	0.36
No	0.49	0.43	0.40	0.38	0.39	0.38	0.38	0.33	0.33	0.33
Cancer										
Yes	0.57	0.49	0.40	0.46	0.44	0.40	0.41	0.40	0.38	0.38
No	0.51	0.44	0.40	0.38	0.39	0.38	0.38	0.34	0.33	0.33

Table S2. Standardized geometric mean of urinary cadmium levels (in μg/g creatinine).

Group	1999–2000	2001–2002	2003–2004	2005–2006	2007–2008	2009–2010	2011–2012	2013–2014	2015–2016
Overall	0.34	0.33	0.32	0.29	0.30	0.29	0.28	0.23	0.24
Sex									
Men	0.29	0.27	0.27	0.25	0.25	0.24	0.23	0.19	0.19
Women	0.39	0.38	0.37	0.32	0.34	0.34	0.33	0.27	0.28
Smoking status									
Never smoker	0.26	0.26	0.25	0.23	0.24	0.24	0.23	0.18	0.19
Former smoker	0.38	0.35	0.36	0.30	0.30	0.32	0.31	0.26	0.27
Current smoker	0.61	0.56	0.53	0.50	0.51	0.52	0.43	0.41	0.40
Chronic kidney disease									
Yes	0.38	0.33	0.36	0.31	0.34	0.35	0.32	0.25	0.26
No	0.34	0.33	0.31	0.29	0.29	0.28	0.27	0.23	0.24
Hypertension									
Yes	0.34	0.33	0.32	0.29	0.31	0.30	0.28	0.24	0.25
No	0.35	0.33	0.31	0.31	0.31	0.29	0.28	0.24	0.25
Diabetes									
Yes	0.35	0.35	0.32	0.31	0.30	0.30	0.27	0.24	0.25
No	0.34	0.33	0.32	0.30	0.30	0.29	0.28	0.24	0.24
Cardiovascular disease									
Yes	0.44	0.34	0.40	0.34	0.40	0.33	0.30	0.34	0.30
No	0.33	0.33	0.31	0.29	0.29	0.29	0.28	0.23	0.24
Coronary heart disease									
Yes	0.43	0.34	0.38	0.39	0.44	0.35	0.32	0.36	0.35
No	0.34	0.33	0.32	0.29	0.29	0.29	0.28	0.23	0.24
Stroke									
Yes	0.58	0.41	0.46	0.38	0.44	0.33	0.36	0.35	0.26
No	0.34	0.33	0.32	0.29	0.29	0.29	0.28	0.23	0.24
Heart failure									
Yes	0.46	0.46	0.55	0.36	0.40	0.37	0.32	0.28	0.42
No	0.34	0.33	0.32	0.29	0.30	0.29	0.28	0.23	0.24
Cancer									
Yes	0.42	0.37	0.31	0.35	0.33	0.28	0.34	0.26	0.24
No	0.37	0.35	0.32	0.28	0.29	0.30	0.28	0.24	0.24
